# Supplementary material for: Modeling tissue-scale ciliary transport and mixing in three-dimensional Newtonian flow
Source: PLoS Comput Biol. 2025 Dec 29;21(12):e1012766. doi: 10.1371/journal.pcbi.1012766 (PMC12779159; doi:10.1371/journal.pcbi.1012766)
Supplement: Attachment — Submitted filename: RebuttalCiliaflow.pdf [file pcbi.1012766.s002.pdf]

Response to Reviewers: Modeling tissue-scale ciliary transport and mixing in three-dimensional Newtonian flow

Ling Xu, Pejman Sanaei, and Yi Jiang

We sincerely thank the reviewers for their thorough reading and constructive feedback. These insightful comments have significantly improved the manuscript. Below, we provide a detailed, point-by-point response to each reviewer's comments, show in red.

Revisions made to the manuscript are highlighted in blue. We have made substantial additions and clarifications throughout the manuscript, particularly regarding the quantitative analysis of mixing, hydrodynamic interpretation of emergent flow structures, and the limitations of our model in approximating physiologic mucociliary clearance.

Review 1

Strengths:

The motivation is clear and the paper is well-written. Experimental results on ciliary sheets are an emerging area, with intrinsic interest due to the relevance of pumping in mucus clearance.

The technical work is high-quality. I agree with the authors' computational approach. IB with rigid rotors is a good approximation for this system, and I like the trick of modifying the effective length, in order to account for the asymmetry of the forward and return stroke. I do not think that a finer level of modeling detail (fluid-structure, force feedback) is necessary for this problem scale.

The authors pick most of their modeling parameters (like spacing between cilia) based on published values, and they vary others over biologically-relevant scales, such as the distances between cilia clusters.

We thank the reviewer for the thoughtful and encouraging remarks. We are especially grateful for the recognition of the clarity of motivation, the quality of the technical work, and the appropriateness of rigid rotors. We appreciate the reviewer's support of our modeling choices, including the use of an effective length to capture stroke asymmetry, and the biologically informed parameter choices.

While I like the problem choice and approach, I'm not as excited about the results, which could be richer and thus more informative for biological systems.

1) The description of the mixing results is overall qualitative, in terms of the number of "swirls" and their relative placement. There are several schematics of flow fields, which are informative, but which would be clearer if they demonstrated an overall scaling trend. I would be interested in a more holistic quantitative measure of mixing quality. The MSD doesn't appear to be sensitive to the qualitative changes in mixing effects that the authors report. The complex 3D mixing dynamics in the full simulations shown in Fig. 5 is a phenomenon worth exploring more deeply.

We thank the reviewer for highlighting the importance of a more detailed and quantitative analysis

of mixing behavior. In response, we have made substantial revisions to deepen the hydrodynamic interpretation, quantify mixing more rigorously, and more clearly demonstrate how mixing emerges and scales in this system.

We agree that MSD is not a good order parameter for mixing and dispersion. We have therefore replaced MSD with a more sensitive and mechanically meaningful metric, the mixing number, previously used in Stone & Stone (2005) and Ding et al. (2014),

$$m = \left(\prod_{i=1}^{N_1} \min_{1 \leq j \leq N_2} \left| \mathbf{z}_i - \mathbf{z}_j \right|^2 \right)^{1/N_1} .$$

This mixing number provides a more direct measure of the evolving vertical proximity between initially segregated particle groups than MSD. The new Fig. 9b and Fig. 22e show the mixing number decreases over time, indicating progressive vertical mixing. At later times, some trajectories show a slight increase in the metric, which is consistent with particle dispersion into the open half-space of the simulation domain, expected under our boundary conditions and model setup.

Main changes:

- Removed all MSD-based figures and discussions.
- Added mixing number as a new quantification for vertical particle evolution and new figures (Fig 9 at tissue-scale and Fig 22 at three-cilia cluster scale) for vertical mixing.
- New discussions around Fig. 9 and Fig 22 (Pages 7-11).

2) I would prefer a more hydrodynamic analysis of the simulation results. From a hydrodynamics perspective, the ciliary carpet injects vorticity into the flow at the patch tips. These vorticity patches appear to combine across scales, giving rise to larger-scale vorticity patches away from the cilia tips. Can we say something more detailed about the relevant scales for mixing and clearance for this system?

We thank the reviewer for this excellent suggestion.

From a hydrodynamic standpoint, the ciliated surface acts as a distributed vorticity source. Each cilium or ciliary cluster generates localized shear near the epithelial surface, injecting vorticity into the surrounding fluid. These vorticity patches initially form at the scale of the cilia clusters ($\approx 10\mu m$), but as they advect and interact, they merge into larger-scale flow structures. At low density, vorticity remains localized and uncoordinated, limiting long-range transport. As ciliary density increases, vorticity patches interact hydrodynamically, leading to constructive interference and large-scale vortex merging. This multiscale interaction cascades upward in scale and contributes to enhanced advection, spatial homogenization, and vertical mixing, in the absence of turbulence. These trends are consistent with low-Reynolds-number mixing theory, where chaotic advection arises from deterministic, structured flows rather than inertial instabilities. Our simulation results show that such interactions lead to emergent mesoscale flow domains (≈ 30–$100\mu m$), consistent with the observed swirl diameters (Fig. 5, bottom row). This multiscale vorticity organization facilitates both directed transport and local mixing by enhancing stretching and folding of particle trajectories. The increase in swirl size and coherence with ciliary density suggests a scale-dependent mechanism: denser cilia arrays produce stronger and more coherent vorticity that propagates farther before dissipating, thus extending the effective transport range.

We have significantly revised the paper to highlight the hydrodynamic analysis. We added new Figs 10, 18, and 24, measuring averaged velocity field and vorticity field to show the hydrodynamic

interactions between vorticity patches and the merging coherent regions of vorticity as ciliary density increases, with and without metachrony.

List of changes to address this comment.

- New figure 10: for synchronized cilia beating, we show trajectories of selected particles from four regions in the domain, average velocity field over one 2π-revolution cilia beating cycle, and vorticity on the x-y plane, at three ciliary densities $\nu = 0.1, 0.2, 0.4$.
- New figure 23: for asynchronized cilia beating, we show average velocity field over one full cilia beating cycle with phase lags $\phi_0 = 2\pi/3, \pi/3, \pi/3$, and $2\pi/3$, respectively.
- New figure 18 shows the vorticity fields for three cilia clusters with varying spacing, demonstrating that $D_c = 5D$ is when the isolated vortices start to interact and enhance transport.
- We have added the above discussions from a hydrodynamic perspective in Results and Discussion.

3) I'm a bit concerned that the "swirls" are a transient effect, particularly because they appear to coarsen over time. Does the flow reach a statistical steady-state, where fluctuations in vorticity, scalar dispersion, etc have a well-defined average? This would help differentiate the effects of spacing/placement conditions.

We thank the reviewer for raising this insightful comment. The "swirls", as visualized by tracer particles, do indeed coarsen over time. This coarsening is due to the cumulative advection of particles (integral of velocity over time) and not an intrinsic change in the underlying hydrodynamics. As particles move with the fluid, they leave their initial regions and merge into larger-scale domains.

To assess whether the flow pattern reaches a statistically steady state, we analyzed the time-averaged velocity fields over full ciliary beating cycles, as in the new Figure 24. These fields remain consistent in both structure and scale across time, indicating that the underlying vorticity field generated by the ciliary carpet reaches a periodic, statistically steady regime.

The newly added Figure 18 shows the stable vorticity fields, demonstrating the hydrodynamic interaction between the ciliary clusters and the optimal spacing for coordinated transport.

We clarified the scales of swirls vs vorticity field and their connection to the optimal spacing for directed transport.

4) For the problems of pumping under synchrony and metachronal waves, the simulation setting seems simplified compared to the more elaborate random X-Y placement of patches in the earlier results (Fig 5). Enhanced transport due to high cilia density is not surprising in itself, since additional cilia inject more power into the flow. I'd be more curious how we might understand this phenomenon in a more complex geometry, particularly in light of the complex eddies that the authors observe when the cilia patches are placed randomly?

We agree that enhanced transport under synchronized beating at high ciliary density is expected due to increase power input (Figs 5 & 6). The metachronal wave introduces richer dynamics that cannot be attributed to density alone. To address this question, we have performed new simulations of the metachronal waves-driven flow fields at the tissue scale. In the revised manuscript, we now compare the time-averaged velocity fields over a beating cycle with (new Fig 24) and without (new Fig. 10d-f). Our results show that symplectic waves (where wave travels in the same direction of the forward/power stroke, $\phi_0 < 0$) lead to coherent merging of local vortices at higher densities, which suppresses eddies and enhances directed flow. In contrast, antiplectic waves (where the wave

travels in the direction of the backward/recovery stroke, $\phi_0 > 0$) lead to destructive interference between neighboring vortices, reducing coordination, and increase mixing.

Regarding the role of geometry complexity, our random x-y placement of cilia patches is intended to mimic the in vivo heterogeneity observed in multiciliated tissues, such as the mouse trachea (Ramirez-San Juan et al., Nature Physics 2020). That work, which we now reference and discuss, demonstrated using a hydrodynamic model that spatial disorder in patch arrangement can enhance clearance due to the emergence of large-scale coordinated flow. Our simulations build on that insight to explore how cilia density and metachrony interact in disordered cilia carpets. We have clarified these points in the revised manuscript.

Main changes in response to this question include

- We added new simulations of cilia-driven fluid flow at the tissue scale with metachrony for three ciliary densities. The time-averaged velocity fields over a cilia beating cycle show symplectic waves lead to coherent merging of local vorticies and enhance transport, while antiplectic waves lead to destructive interference between vortices and reduce directed flow.

- We clarified that the random spatial distribution of ciliary patch was mimicking real airway tissue and tissue culture.

- We added the reference to Ramirez-San Juan et al., Nature Physics 2020 and Kanale et al., PNAS 2022, discussed their results on geometrical disorder in ciliary patch distribution enhances mucociliary clearance, and emphasized our work is on the roles of cilia density and metachronal coordination in transport and mixing in a disordered ciliary carpet.

1) I'm confused regarding the finding that metachronal waves inhibit transport. Doesn't this depend very sensitively on the beating rate, phase offsets, spacing, flow rate, etc?

Thank you for the insightful comment. We have extensively expanded the results to show that metachronal waves in our model mostly inhibit transport. This observation depends on the phase lag: some symplectic waves can promote transport, while most metachronal coordination impedes transport, some antiplectic waves even reverse flow direction. The trend holds true for spacing (new Figure 21).

We chose a cilia beating rate as provided by experiment (Sanderson & Sleigh, 1981). As the cilia beating rate can vary across different regions of the human airway and under different physiological or pathological conditions, we have also tried to vary the beating rate in the three-cluster simulations and found no significant difference in transport in the $x-$direction (Fig. 1.)

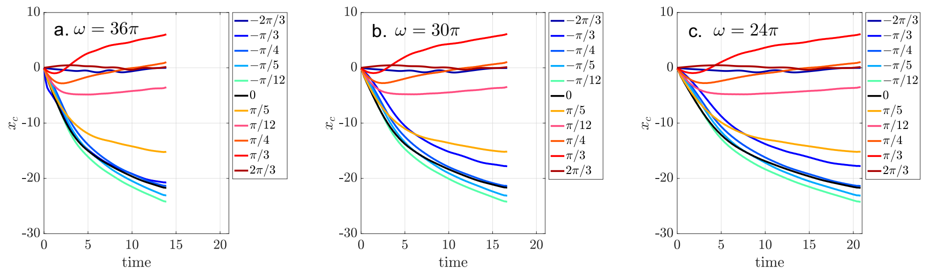

Figure 1: Particle transport (center of mass of particles in the x-direction) in three cilia clusters with spacing $D_c = 3D$ for three angular velocities, $\omega = 36\pi, 30\pi$, and 24π, after 400 cilia beating revolutions.

2) Are all cilia in all clusters always aligned (pumping the same direction)?

Cilia in all clusters are aligned in synchronized beating. When we simulate metachronal waves, cilia are organized in columns of three with a phase lag between columns. We have clarified this point in the model description.

Under "Tissue-scale cilia clusters", we added "When we simulate metachronal waves, cilia in each columns of three are synchronized with a phase-lag ϕ_0 between the columns of cilia." on page 6.

3) Why is enhanced mixing or particle dispersion desirable in this system? In a biological system such as the lungs, is net transport the only readout that matters?

The mixing and dispersion are desired for delivery of aerosolized medicines or vaccines via nose or mouth. We have clarified this point on page 9.

Page 9: we added "In the human airway, effective mucociliary clearance relies on preventing pathogenic particles from reaching the epithelial surface by keeping them trapped in the mucus layers. In contrast, for aerosolized drug delivery, the desirable outcome is the opposite: rapid transport of therapeutic agents toward the cells. Therefore, understanding how cilia motion drives vertical movement is important to both clearance and delivery. "

4) The authors observe a bias towards counter-clockwise rotation, and the net flow has a slight diagonal drift. What is causing this effect? In my reading, I didn't see what breaks the symmetry in the system.

The cilia rotation is not symmetric (see Fig 2 and equation 2). The cilium length is fixed during the effect stroke ($\theta \in [0, \pi]$) and is shorter (and as a function of angle) during the recovery stroke ($\theta \in [\pi, 2\pi]$). We clarified this point in the model description.

On page 5, right after equation 2, we changed the text to be more clear "... where L is longer during the forward stroke than the backward stroke." As shown in Fig.2(c), the trajectory of the rod tip is not circular on the (x, y)-plane due to the variation of both θ and the rod length."

5) Are cilia clusters known to be distributed randomly? Or do they typically appear in evenly-spaced rows, like those considered in the metachronal wave and synchronization results?

Yes, airway tissues from human and animal models as well as reconstructed airway tissues all show randomly distributed cilia clusters, as in Sanderson and Sleight (1981), Khelloufi et al. (2018), Ramirez-San Juan et al. (2020). This point has been clarified in the revised text.

Page 6: "Previous models of ciliary carpet have considered a uniform ciliar array (e.g., [25]). Our model explicitly considers the random x-y placement of cilia clusters to mimic the experimental observed heterogeneity in ciliated cell distribution in mouse trachea [40] and reconstituted human airway tissue cultures [5, 8, 9]."

Reviewer 2

This paper is extremely well written, providing a significant step forward beyond results available in the literature on an important problem in respiratory physiology: mucociliary clearance of the barrier fluid coating the respiratory tract.

The paper summarizes "target data and information" with respect to existing experimental and clinical data on cilia propulsion of mucus, and uses that data as a basis for their modeling approach. This is "all good" and represents a nice contribution to the literature.

We thank the reviewer for the generous and encouraging feedback. We are glad that the reviewer finds our manuscript well written and a valuable contribution to the field of mucociliary clearance

and respiratory physiology. We especially appreciate the recognition of our effort to ground the modeling approach in relevant experimental and clinical data. This affirmation is motivating and has strengthened our commitment to refining the clarity and scientific rigor of the work.

However, there are two essential missing ingredients in their model of mucociliary clearance.

One: The authors make the assumption that has been made by the preponderance of the historical literature on modeling of cilia propulsion of mucus. This history includes cilia forcing of the mucosal barrier at the single cilium scale as well as attempts to explain synchronization of cilia and propulsion at the cellular ($\sim$ 200 cilia) and tissue scales. Namely, the foundation of this paper and most previous modeling of cilia propulsion of mucus is based on the assumption that the mucus layer is an incompressible viscous fluid. Mucus is not a viscous fluid; it is an entangled polymer solution whose properties are not remotely approximated by the Stokes equations as done here (and others in previously published work).

Two: The cilia beat stroke consists of a power stroke in which the cilium is approximately rigid and rod-like and penetrates the mucus layer by one micron at the peak of the power stroke, but the return stroke is quite curved and does not make contact with the mucus layer. That stroke is far from the model in this paper, and the flows generated will almost surely be wildly different even in a viscous fluid. So the beautiful modeling done in this paper does not shed light on the transport of mucus in the annular "fluid layer" between the cellular tissue and the air core.

That said, to their credit, there are several important details present in the paper that are extensions of what has been done previously. So I am happy to support publication if the title and the claims are walked back to something along the lines of: "modeling of ciliary transport of viscous fluids in airway-like geometries". Then one can say what is new here, and that the results need to be extended significantly to "solve" the real problem of physiologically relevant mucociliary clearance. Otherwise readers might think this paper is relevant to the human respiratory tract – and with all due respect, it is not! I can say, with absolute certainty, that the flows generated by beating cilia at the cellular and tissue scale are wildly different for viscous fluids and mucus. It is misleading to suggest otherwise.

Critiques well taken. In response, we clarify that our model extends a commonly used computational simplification of treating the fluid between the air and epithelium as a simple Newtonian fluid. We recognize that this approximation does not account for the multi-layered fluid structure or the non-Newtonian, viscoelastic nature of mucin network in the airway mucus. We have substantially revised the manuscript to reduce the references to "mucus" where inappropriate and highlighted the differences between the ideal Newtonian fluid and the airway mucus. Major changes appear in the Title, Abstract, Introduction, and Discussion, as detailed below.

We have made the following changes:

Title: "Modeling tissue-scale ciliary transport and mixing in three-dimensional Newtonian flow"

Abstract: "We present three-dimensional (3D) simulations of ciliary-driven mixing and transport of a Newtonian fluid as an approximation of mucus at the tissue scale. We investigate the influence of ciliary density, cilia cluster spacing, and metachrony on fluid mixing and transport. ... ... These findings provide useful insight into generic principles of cilia-driven transport in viscous fluids and may inform bio-inspired system design, while further work is needed to extend this work to physiologically realistic mucus transport."

Introduction: "In all these mathematical models, mucus has been treated as an incompressible viscous fluid. However, airway mucus is a sticky gel composed of mucin and periciliary-glycocalyceal layer [35]. While these studies collectively underscore the importance of computational models in unraveling the nuances of cilia-driven flow, it is important to remember the difference to physiolog-

ically accurate mucociliary system in airway.

The objective of this study is to address how ciliary density and distribution impact the mucociliary transport and mixing at the tissue level. We adopt the regularized Stokeslet method and assume an incompressible Newtonian fluid to approximate mucus flow, following standard modeling practices [36]. We acknowledge that mucus is a complex viscoelastic gel and that Stokes flow models do not capture its polymeric structure or time-dependent rheology. However, this assumption allows us to isolate and analyze key geometric and kinematic effects of ciliary distribution and coordination.

Discussion: "Our idealized constitutive model of mucus and cilia motion provides a computational platform for examining the fluid dynamic consequences of ciliary density, spacing, and metachrony in simplified fluid environments. However, we emphasize that our simulations are not designed to replicate physiologically accurate mucociliary clearance in the human airway. First, mucus is a viscoelastic gel, its polymeric network property deviates substantially from the Newtonian fluid assumption used here. Second, we adopt a simplified representation of cilia as rotating rigid rods, which does not capture the asymmetric power and recovery strokes observed in vivo. These limitations mean that the modeled flows likely differ qualitatively from actual mucus transport. Nevertheless, the insights obtained from this idealized system may still inform the development of artificial cilia arrays and inspire hypotheses for future models that include non-Newtonian fluid properties and fully resolved ciliary kinematics."

Furthermore,the diffusion of small molecule concentrations performed in this paper will not be relevant to airway surface liquids and drug delivery .... the advection-diffusion will be strongly dominated by the correct 3D advection velocity field, on the upside, small molecule diffusion is not dramatically different since sufficiently small molecules will diffuse normally and not undergo diffusion with memory as large molecules or particles in complex polymeric fluids.

We appreciate the reviewer's comment and apologize for the confusion caused by our wording. To clarify, our model does not model diffusion. We tracked the trajectories of massless passive tracers, and the mean squared displacement (MSD) was used solely as a quantification to access particle dispersion over time. While we compared the resulting MSD curves to simple diffusion and anomalous diffusion trends, this was intended as a qualitative reference rather than a model of molecular diffusion.

To avoid this misunderstanding, we have removed all MSD-based quantification and replaced them with a mixing number that more directly characterizes particle redistribution due to flow. This change has been implemented throughout the manuscript where appropriate.

If the authors state very clearly that this idealized constitutive model of mucus in their modeling platform provides a foundation for extension to complex fluid models of mucus, and an idealized model of the cilium beat stroke, then the reader will understand that the relevance to the respiratory tract is unknown and this paper is giving a preview of what it might actually look like. NOBODY HAS DONE THIS IN 3D SIMULATIONS, even with viscous fluids, SO THE RESULTS IN THIS PAPER ARE AN IMPORTANT CONTRIBUTION! To become more relevant, one will need to derive a good entangled polymer model for mucus, and couple that constitutive equation to the Navier-Stokes or Stokes model as is done in all viscoelastic fluid solvers. This advance – a good continuum scale constitutive equation of respiratory mucus, remains an open problem. There is a significant literature on microrheology of mucus that produces the equilibrium viscoelastic moduli of mucus, but it is still a large step to take that data and embed it into a macroscopic constitutive equation. That is itself an open problem. Another open problem is to use dynamic microscopy to get the full cilium beat stroke shape.

For this reason, the authors cannot be criticized for not modeling physiological mucociliary clearance.....that problem is not going to be solved anytime soon.

Critique acknowledged and appreciated. See response above and the list of changed, especially the paragraphs added to Discussion.

Reviewer 3

The authors present a computational study of cilia-driven flows. The motion of the individual cilium is prescribed and fixed throughout the manuscript. The authors vary the cilia density and distances between patches of cilia. They compute the flow velocity field, using a Stokeslet model, and they follow the motion of passive tracers to quantify mixing. They report transport and mixing by synchronously beating cilia. They report reduced transport by cilia beating in a metachronal fashion, where meta-chronicity is imposed, not emergent, by implementing a phase difference between individual cilia.

We thank the reviewer for this synopsis of our work and these encouraging comments.

The methods utilized here are the same methods used in Ding, Yang, et al. "Mixing and transport by ciliary carpets: a numerical study." Journal of Fluid Mechanics 743 (2014): 124-140. Any online search on the topic of mixing and transport by cilia would have led the authors to this work, yet this work is not cited, despite its immediate relevance to the present study.

In this study, the beating pattern of the individual cilium is different from the beating pattern in Ding et al., 2014 – the later uses a beating pattern reconstructed from actual tracheal cilia, while in this study the authors use an abstraction of nodal-beating cilia. The boundary conditions in this study are also different from the boundary conditions in Ding et al., 2014 – here, the authors focus on small patches of regularly-spaces cilia surrounded by no cilia, while Ding et al., 2014 focused on doubly-periodic arrays of cilia. Other than these two differences, the two studies are nearly identical. This is not a problem had the current study presented novel insights into new physics or new flow patterns facilitated by these differences.

We thank the reviewer for bringing this reference to our attention. We have now cited in the revised paper.

We agree that the work of Ding et al. (2014) shares important methodological elements with ours, particularly in the use of particularly in the use of the regularized Stokeslets method (Ainley et al., 2008) to model flow driven by cilia carpets and in the focus on transport and mixing. However, in addition to the differences in cilia beating patterns and boundary conditions already pointed out by the reviewer, we emphasize that our work differs in several additional ways:

1. Our model is fully 3D, with 3D cilia beating motion and 3D flow, whereas Ding et al. (2014) restricted the cilia beating to 2D. Therefore their flow patterns were intrinsically 2D in the $x - z$ plane. This difference may significantly impact the dynamics of transport and mixing.

2. We have randomly distributed cilia clusters with varying density; they had a uniform cilia carpet. This distinction is important as spatial heterogeneity not only is reality but also has been shown to enhance clearance (Ramirez-San Juan et al., Nature Phys. 2020). Our study explores these effects.

3. While Ding et al. primarily focused on metachrony in periodic cilia carpets, our work studies the impacts of both spatial heterogeneity and metachronal coordination on transport and

mixing. Our results show the transport and mixing as a result of of ciliary density, cluster spacing, and phase lags acting together.

4. Furthermore, our simulations discover an optimal ciliary spacing for enhancing transport.
5. We find that metachrony, in contrast to Ding et al. (2014), generally reduces clearance transport while increasing mixing, particularly in sparse and heterogeneous configurations.

We have extensively revised the paper to clarify these points and to explicitly differentiate our work from previous studies, including Ding et al. (2014).

The work of Loiseau et al, 2020, Nature Physics and Khelloufi et al, 2018, Scientific reports, that report swirling motion in ciliated tissues is not done in intact human tissues, but in highly-reconstructed, in-vitro preparation that focuses on **ciliagenesis** – in the abstract of Loiseau et al, 2020, the authors write "we show on polarized human bronchial epithelia that mucus swirls and circular orientational order of the underlying ciliary beats emerge and grow during ciliogenesis, until a macroscopic mucus transport is achieved for physiological ciliary densities." (bold italic is mine). The current manuscript emphasizes the similarity between a swirling motion in the computational model to these experimental observations but seems to suggest that this behavior is typical in intact functional tissues. Importantly, the manuscript provides no insights into the mechanisms responsible for these flow patterns.

Thank you for this insightful comment. We have significantly revised the manuscript to mor carefully explain the differences between our simplified computational model, reconstructed in vitro tissue models, and the airway tissue. In particular we now explicitly state that the experiments in Loiseau et al. and Khelloufi et al. were performed in developing, in vitro airway epithelia undergoing ciliogenesis. While these systems do not fully represent mature, functional airway tissue, they offer controlled environments to explore how ciliary organization gives rise to mesoscale flow structures, such as mucus swirls. We do not claim that swirling flow is a universal feature of intact tissues, but rather that our model demonstrates how hydrodynamic interactions between distributed ciliary sources can give rise to mesoscale vortex structures—qualitatively consistent with observations in both experimental preparations and recent modeling studies (e.g., Ramírez-San Juan et al., Nat. Phys. 2020).

About new mechanistic insight, we have substantially expanded the discussion and added a new section in the Results that analyzes the hydrodynamic origin of the emergent swirls and their roles in transport and mixing. Each cilium or cluster acts as a localized shear generator that injects vorticity into the surrounding fluid. At low density, vortices remain isolated; at higher density, coherent interactions between vortices drive vortex merging, producing larger-scale flow domains. Under synchrony or symplectic metachrony, vortices merge constructively, enhancing transport. In contrast, antiplectic metachrony leads to destructive interference and diminished forward flow—sometimes even reversing it at large phase lags. These coherent flow domains enhance stretching and folding of particle trajectories, promoting mixing via deterministic low-Reynolds-number mechanisms.

Major changes include:

- A detailed list of changes is included in response to reviewer 2 above on the distinction between our computational model and in vitro and in vivo airway mucociliary clearance.
- In Introduction, added explicit discussion of these in vitro models were undergoing ciliogenesis and do not fully represent, mature functional airway tissue.

- We have cited Vilfan et al., PNAS 2010, Loiseau et al., 2020, Ramirez-San Juan et al., Nature Physics 2020 to position our study within a broader literature.
- We have added to Results and Discussion analyzes the hydrodynamic origin of the emergent swirls and their roles in transport and mixing.

Technically, I find the manuscript lacking in the following aspects:

1) The manuscript is observational. The authors describe flow fields and mixing patterns they obtain computationally but do not discuss the essential mechanisms leading to these observations (e.g., individual cilium beating pattern? Boundary conditions?) nor the implications of these observations to healthy or pathological ciliated tissues.

We appreciate this important observation and agree that clarifying the mechanisms behind emergent flow and mixing behaviors is important. In response, we have substantially revised the manuscript to deepen the hydrodynamic interpretation and explicitly link observed flow structures to the ciliar distribution and underlying ciliary kinematics.

Specifically, we now highlight that each cilium or cilia cluster acts as a localized source of vorticity near the epithelial surface. The asymmetry in the prescribed beat pattern (with a longer effective length during the power stroke and shortened recovery stroke) generates directional shear, which interacts with adjacent flow fields depending on spatial proximity and phase coordination. At low densities, vorticity remains localized, while at higher densities, these patches merge into coherent mesoscale structures, driving advection and mixing. This mechanism is analyzed in new Figures 10, 18, and 23, and further discussed in the Results and Discussion sections.

We also clarify that the observed swirling flows arise not from transient artifacts but from steady-state hydrodynamic interactions. The time-averaged velocity and vorticity fields demonstrate consistent mesoscale structure over multiple cycles, confirming statistical steady states of the flow.

Regarding biological implications, we have revised the Discussion to explicitly distinguish between features of our idealized Newtonian model and actual mucociliary physiology. While we do not model disease states directly, we note that the spatial heterogeneity in cilia density and coordination, which is often disrupted in pathological tissues, is central to our investigation. Thus, our results provide insight into how such parameters could modulate transport efficiency, which may inform hypotheses for future experimental or more biophysically detailed modeling studies.

2) The study of cilia density is technically flawed. From a fluid mechanics perspective, to assess the efficiency of transport and mixing, one would have to either conserve the total hydrodynamic energy imparted to the fluid, or the total ciliary mass. Neither is conserved, nor even discussed. The fact that higher cilia density improves both transport and mixing is a no-brainer: you have more of the same stuff pushing the fluid, you'll get enhanced transport and mixing.

We agree that, under synchronized beating, increased cilia density would naturally lead to greater momentum injection into the fluid and stronger transport and mixing (as shown in Figs 5 & 6). However, our aim is not to show the transport enhancement is surprising under synchrony, but rather to explore how metachronal coordination modulates this effect across different densities. To clarify this point, we have now included new simulations in the revised manuscript, examining flow fields generated by metachronal waves at the tissue scale. Presented in new Fig 23 and compared to the synchrony case in Fig 10d-f, these simulations show that, at low cilia densities, vortices generated by individual clusters remain isolated, producing limited long-range flow. As density increases, the interaction between neighboring vortices becomes more significant and have non-trivial consequences. With symplectic metachrony, these interactions lead to constructive merging of

vortices and enhance direct flow. With antiplectic metachrony, the interactions become disruptive, reducing directed flow in the negative x direction, and can even reverse the flow direction as in the case of three cilia clusters (new Fig 21).

Regarding the issue of energy conservation, we disagree with the comment: we examine the effect of inter-cluster spacing with the same three cilia clusters over the same 400 beating cycles; we also study the effect of metachrony of the same ciliary density. We have clarified this point and expanded the relevant discussion in the revised manuscript.

Major changes include

- New Figure 21 showing the center of mass of tracer particles over 400 beating cycles as a function of inter-cluster spacing and phase lag.
- New Figuer 23: time averaged velocity field as a function of ciliary density and metachrony.

3) The study of transport and mixing by ciliary patches is technically questionable. Modeling patchy-tissues is fundamentally stochastic: the top row in figure 5 is only a single realization. Any conclusion should be based on Monte Carlo simulations and multiple realizations. This aspect is not discussed in the manuscript. There is mention that the results in Fig. 6 are the average of three independent simulations, but we don't know what probability density function, if any, these simulations sampled from. Here, I suggest that the authors read the following work: Kanale, Anup V., et al. "Spontaneous phase coordination and fluid pumping in model ciliary carpets." Proceedings of the National Academy of Sciences 119.45 (2022): e2214413119.

Thank you for highlighting this important point. Indeed, ciliary patch placement in tissue is intrinsically stochastic and that drawing robust conclusions requires considering this variability.

In our tissue-scale simulations, the locations of ciliary clusters are randomly sampled using a uniform distribution over the simulation domain ("randn" function in Mathlab). Each analysis is based on 3 independent realizations of random cluster placements. Fig 5 is a representative simulation to visualize the spatial flow patterns. However, all quantitative analyses, including the center of mass, the mixing number, and Ripley's $K(r)$, are based on averaging across three independent simulations.

We appreciate the reviewer's suggestion to consult the work by Kanale et al. (PNAS 2022), which we have now read and cited. That study investigates spontaneous phase coordination in model ciliary carpets and emphasizes the role of hydrodynamic coupling and disorder. While our study does not model phase dynamics explicitly, we share the interest in how spatial disorder and metachronal waves influence emergent transport. We have incorporated a discussion of these distinctions in the revised manuscript to situate our results in the broader context of ciliary fluid dynamics.

Major changes include

- We have now clarified the sampling procedure in the revised manuscript (page 5).
- We have added to Discussion that additional realizations may strengthen the statistical robustness of our findings.

4) The statement that "metachronal wave(s) inhibit transport" is the most provocative and potentially interesting statement in the entire work. But there is no exploration of the mechanisms leading to this observation – is this specific to the beating pattern employed here? It is well-known

that metachronal coordination in cilia lead to improved transport. What is different here? is it the nodal-beating pattern of the individual cilium? What are the effective and recovery stroke directions in this model of the individual cilium? what does transport and mixing look like for an individual cilium? How does the ciliary array and metachronal coordination between individual cilia change that?

Thank you for the insightful comment. We have more carefully conducted further simulations at the tissue scale with metachronal waves and analyzed the flow and mixing as a result of metachrony. The main difference between our model and the previous publications include:

1. Local metachronal coordination: In our model, the metachronal wave propagates along the $x-$direction within each cilia cluster, with a fixed phase-lag ϕ_0 between adjacent cilia columns. Our cilia only occupy a truncated portion 8 μm of the wavelength. Between cilia clusters, especially at low ciliary densities, the inter-cluster spacing is more likely incompatible with the metachronal wavelength, leading to destructive interference.

2. Open boundary condition: Our computational domain is an open half-plane, enabling particles to move freely away from cilia clusters without spatial constraints over time. In addition, the resulting fluid flow will be different due to the influence of boundary conditions.

3. Definition of transport: We measure the clearance transport by the center of mass of the tracer particles. With the different boundary conditions, this measure is different from the total displaced volume over one ciliary beating cycle $\int_0^T Q_v dt$ in previous publications (e.g., Ding (2014) and Chateau et al (2017, 2018).

To clarify the difference, we look at the formulation of the global volume rate,following Chateau et al (2017) (equation 3.1). The global volume rate, $Q_v = N_z \dfrac{U^* dx^2}{L^2}$, is defined over a unit volume of size $(1 \times 1 \times n_z)$, where $U^* = U^{\text{av}}/U^{\text{ref}}$ is the dimensionless velocity ratio, $U^{\text{av}} = \sum_{i,j,k} u_{ijk}/(n_x n_y n_z)$ is the average fluid velocity inside the simulation domain, and $U^{\text{ref}} = (n_x/N_{\text{cil}})/T$ is the reference velocity, with N_{cil} the total number of cilia and $T = 2\pi/\omega$ the ciliary beat period.

This global volume rate Q_v cannot be not properly defined in our open boundary conditions!

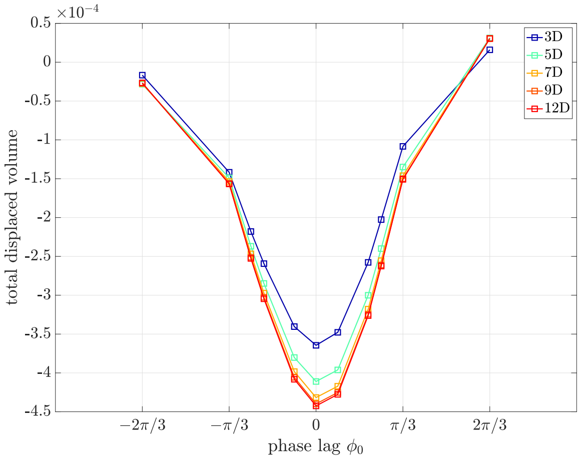

Figure 2: Total volume displacement for (a) 3-ciliar cluster system across various inter-cluster spacing ($D_c = 3D, 5D, 7D, 9D$, and $12D$, and (b) tissue scale across various ciliary density $\nu = 0.1, 0.2$, and 0.4.

Nevertheless, we plot total displacement volume $\int_0^T Q_v dt$ in Figure 2. The average velocity is evaluated using a big box of $[-100, 100] \times [-16, 16] \times [0, 10]\mu m$. We see it shows a different picture

from new Figure 21 (center of mass).

1. $D_c = 3D$, $\phi_0 = -\pi/12$ no longer has stronger transport than synchrony, and $\pi/3$ no longer reverses flow.

2. $D_c = 5D$ is no longer optimal in clearance transport.

The discrepancy arises because $\int Q_v dt$ is not well defined in the open half space, as indicated earlier; Moreover, $\int Q_v dt$ measures total displaced volume while the center of mass of particles measures net transport. We therefore cannot compare directly with the previously published result.

We have added this discussion in Discussion.
