# Supplementary material for: Modeling tissue-scale ciliary transport and mixing in three-dimensional Newtonian flow
Source: PLoS Comput Biol. 2025 Dec 29;21(12):e1012766. doi: 10.1371/journal.pcbi.1012766 (PMC12779159; doi:10.1371/journal.pcbi.1012766)
Supplement: Attachment — Submitted filename: CiliaflowRebuttal2.pdf [file pcbi.1012766.s003.pdf]

Response to Reviewers 1 and 2:

We sincerely thank Reviewers 1 and 2 for their support of our revision and recommendation for publication.

Response to Reviewer 3:

I have read the revised manuscript "Modeling tissue-scale ciliary transport and mixing in three-dimensional Newtonian flow" and I have read the authors response to my comments. I appreciate the authors' efforts to revise their work and cite the relevant work (e.g., "Mixing and transport by ciliary carpets: a numerical study"). Note that in their response letter, the authors mischaracterize the study of Ding et al to be 2D – the fluid motion is 3D but the cilia beating is 2D.

We thank Reviewer 3 for taking the time to reread the revised manuscript. We also appreciate the correction regarding our earlier characterization of Ding et al.'s model. Indeed, while the cilia beating pattern was confined to 2D, their simulations resolved fully 3D flow, as shown in their Figure 3. We have corrected this misstatement in our current rebuttal and appreciate the clarification.

My major concerns about the study still hold. The study offers no new methods and no deep insights into the mechanisms that lead to the swirling motion in this set-up.

While we respect the reviewer's continued reservations, we reiterate and expand on the key distinctions and new insights contributed by our work:

Key distinctions from Ding et al. (2014): .

1. Fully 3D cilia beating and flow. Our model incorporates 3D cilia beating that generates fully 3D time-dependent flow fields. This differs fundamentally from prior studies with planar (2D) beating. The resulting vertical transport and mixing are critical for understanding clearance and penetration in physiological contexts, dynamics that differ significantly from those driven by 2D beating.

2. Tissue-scale spatial heterogeneity. Unlike idealized periodic arrangement of cilia carpets, we explore tissue-scale, non-uniform distribution of ciliary clusters with varying densities. Spatial heterogeneity is not only physiologically relevant but has been shown to enhance clearance (Ramirez-San Juan et al., Nature Phys. 2020).

3. Extended temporal scale. We simulate hundreds of beating cycles (400 cycles), far more than the typical one cycle simulations, which allow us to study long-term transport and mixing behavior, swirl coarsening, vortex merging, and mixing saturation, phenomena not accessible in shorter simulations.

4. New insights into transport and mixing mechanisms:

   (a) We quantify how ciliary density, spatial arrangement, and metachronal coordination impact both horizontal transport and vertical mixing.

   (b) Our simulations identify an optimal ciliary spacing for enhancing directional transport.

(c) We find that metachrony, in contrast to Ding et al. (2014), generally reduces clearance transport while increasing mixing, particularly in sparse and heterogeneous configurations. Symplectic waves lead to coherent merging of local vortices at higher densities, which suppresses eddies and enhances directed flow. In contrast, antiplectic waves lead to destructive interference between neighboring vortices, reducing coordination, and increase mixing.

Importantly, given the stochastic nature of the input, the study should employ rigorous Monte Carlo simulations and appropriate statistical metrics to draw meaningful conclusions, rather than relying on isolated, single-run observations. Add to this that the link to physiological function of such swirling fluid motion is weak, I am not very enthusiastic about this work. I leave the final decision to the editor.

We respectfully clarify a misunderstanding regarding our use of randomness. Our results are not based on isolated, single-run simulations. At each ciliary density, we performed three independent realizations with randomly distributed cilia patches and tracked outcomes over 400 full beating cycles. Across these runs, the trends in mixing and transport were consistent and robust, as documented in Figures 6, 7, and 9. We do not expect these observations to change significantly with additional random realizations of cilia of the same densities. Given that our primary focus is on the effects of density and coordination, not on a full stochastic exploration of spatial arrangements, full Monte Carlo simulations are not required. We believe our sampling strikes a practical balance between robustness and computational cost.

While the exact functional implications of swirl dynamics in vivo remain an open area, our study provides mechanistic insights into how global clearance and local mixing emerge from hydrodynamic interactions. These are central to understanding processes such as mucus clearance and drug delivery in the airway.

We have revised figures 6, 7, and 9 to include standard deviations based on 3 independent random realizations for each ciliary density. These additions clearly shows that, despite variability among realizations, the overall trend remains the same.
