# Supplementary material for: Modeling tissue-scale ciliary transport and mixing in three-dimensional Newtonian flow
Source: PLoS Comput Biol. 2025 Dec 29;21(12):e1012766. doi: 10.1371/journal.pcbi.1012766 (PMC12779159; doi:10.1371/journal.pcbi.1012766)
Supplement: Attachment — Submitted filename: ResponseFinal.pdf [file pcbi.1012766.s004.pdf]

Modeling tissue-scale ciliary transport and mixing in three-dimensional Newtonian flow

Ling Xu, Pejman Senaei, Yi Jiang

Response to reviewer

In the last round of review, all reviewers indicated acceptance pending one remaining item: making our code repository public and citing it with a DOI. We have completed this request. The repository is now public and archived on Zenodo with a DOI: GitHub repo: https://doi.org/10.5281/zenodo.17560085

(https://github.com/Jiang-Lab/Mucociliary_Clearance-Tissue_Scale_model).

We have done so.

We have also updated all figures such that they satisfy PLoS Comp Bio figure requirements.

We updated the manuscript to include a data availability statement, and all updated figures.
