# Supplementary material for: Modeling tissue-scale ciliary transport and mixing in three-dimensional Newtonian flow
Source: PLoS Comput Biol. 2025 Dec 29;21(12):e1012766. doi: 10.1371/journal.pcbi.1012766 (PMC12779159; doi:10.1371/journal.pcbi.1012766)
Supplement: Attachment — Submitted filename: Review for PLOS Computational Biology.docx [file pcbi.1012766.s001.docx]

Review for PLOS Computational Biology (PCOMPBIOL-D-24-02256)

I read the manuscript entitled “Modeling mucociliary mixing and transport at tissue scale" by Dr. Yi Jiang and colleagues that was submitted for publication in PLOS Computational Biology.

The authors present a computational study of cilia-driven flows. The motion of the individual cilium is prescribed and fixed throughout the manuscript. The authors vary the cilia density and distances between patches of cilia. They compute the flow velocity field, using a Stokeslet model, and they follow the motion of passive tracers to quantify mixing. They report transport and mixing by synchronously beating cilia. They report reduced transport by cilia beating in a metachronal fashion, where meta-chronicity is imposed, not emergent, by implementing a phase difference between individual cilia.

I find that the manuscript does not bring any substantial new knowledge nor substantial new methods compared to existing computational work on cilia.

The methods utilized here are the same methods used in Ding, Yang, et al. "Mixing and transport by ciliary carpets: a numerical study." *Journal of Fluid Mechanics* 743 (2014): 124-140. Any online search on the topic of mixing and transport by cilia would have led the authors to this work, yet this work is not cited, despite its immediate relevance to the present study.

In this study, the beating pattern of the individual cilium is different from the beating pattern in Ding et al, 2014 -- the later uses a beating pattern reconstructed from actual tracheal cilia, while in this study the authors use an abstraction of nodal-beating cilia. The boundary conditions in this study are also different from the boundary conditions in Ding et al, 2014 – here, the authors focus on small patches of regularly-spaces cilia surrounded by no cilia, while Ding et al, 2014 focused on doubly-periodic arrays of cilia. Other than these two differences, the two studies are nearly identical. This is not a problem had the current study presented novel insights into new physics or new flow patterns facilitated by these differences.

The work of Loiseau et al, 2020, Nature Physics and Khelloufi et al, 2018, Scientific reports, that report swirling motion in ciliated tissues is not done in intact human tissues, but in highly-reconstructed, in-vitro preparation that focuses on ciliagenesis – in the abstract of Loiseau et al, 2020, the authors write “we show on polarized human bronchial epithelia that mucus swirls and circular orientational order of the underlying ciliary beats emerge and grow *during* ***ciliogenesis***, until a macroscopic mucus transport is achieved for physiological ciliary densities.” (bold italic is mine). The current manuscript emphasizes the similarity between a swirling motion in the computational model to these experimental observations but seems to suggest that this behavior is typical in intact functional tissues. Importantly, the manuscript provides no insights into the mechanisms responsible for these flow patterns.

Technically, I find the manuscript lacking in the following aspects:

1. The manuscript is observational. The authors describe flow fields and mixing patterns they obtain computationally but do not discuss the essential mechanisms leading to these observations (e.g., individual cilium beating pattern? Boundary conditions?) nor the implications of these observations to healthy or pathological ciliated tissues.
2. The study of cilia density is technically flawed. From a fluid mechanics perspective, to assess the efficiency of transport and mixing, one would have to either conserve the total hydrodynamic energy imparted to the fluid, or the total ciliary mass. Neither is conserved, nor even discussed. The fact that higher cilia density improves both transport and mixing is a no-brainer: you have more of the same stuff pushing the fluid, you’ll get enhanced transport and mixing.
3. The study of transport and mixing by ciliary patches is technically questionable. Modeling patchy-tissues is fundamentally stochastic: the top row in figure 5 is only a single realization. Any conclusion should be based on Monte Carlo simulations and multiple realizations. This aspect is not discussed in the manuscript. There is mention that the results in Fig. 6 are the average of three independent simulations, but we don’t know what probability density function, if any, these simulations sampled from. Here, I suggest that the authors read the following work: Kanale, Anup V., et al. "Spontaneous phase coordination and fluid pumping in model ciliary carpets." *Proceedings of the National Academy of Sciences* 119.45 (2022): e2214413119.
4. The statement that “metachronal wave(s) inhibit transport” is the most provocative and potentially interesting statement in the entire work. But there is no exploration of the mechanisms leading to this observation – is this specific to the beating pattern employed here? It is well-known that metachronal coordination in cilia lead to improved transport. What is different here? is it the nodal-beating pattern of the individual cilium? What are the effective and recovery stroke directions in this model of the individual cilium? what does transport and mixing look like for an individual cilium? How does the ciliary array and metachronal coordination between individual cilia change that?

Overall, the authors implemented a computational framework for solving for cilia-driven flows. This computational framework is not novel. They reported observations, based on their computations, without deeper explanation of the physical mechanisms leading to these observations. Besides qualitative statements, the authors did not connect their observations to actual ciliated tissues. Given these major shortcomings, I don’t recommend the manuscript for publication in PLOS Computational Biology.
